# Supplementary figures and images for: Seasonal dynamics of terrestrial vertebrate abundance between Amazonian flooded and unflooded forests
Source: PeerJ. 2018 Jun 27;6:e5058. doi: 10.7717/peerj.5058 (PMC6026452; doi:10.7717/peerj.5058)

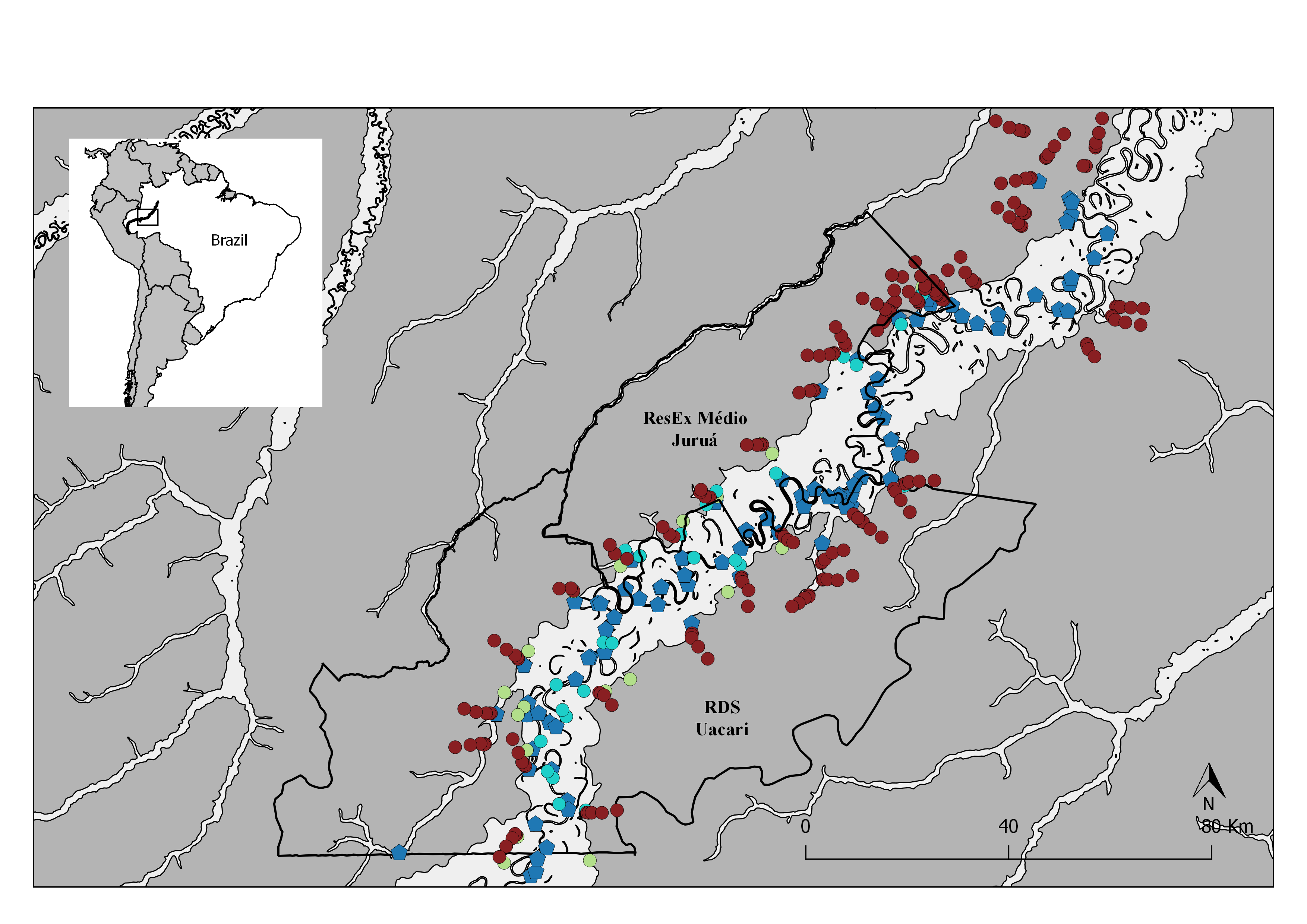

Supplement: Figure S1 — Map inset shows the geographic location of the Juruá river and study region. The boundaries of the RESEX Médio Juruá and RDS Uacari are outlined in black. Seasonally flooded forests and terra firme forests are represented in light and dark gray respectively. Solid red circles represent camera trap stations (CTS) deployed radiating inland into terra firme forest (sample design 1). Green and aqua circles represent CTS deployed at terra firme forest sites near forest habitat boundaries along the várzea interface and far into várzea forest, respectively (sample design 2). Blue pentagons represent the location of human settlements. [file peerj-06-5058-s005.png]

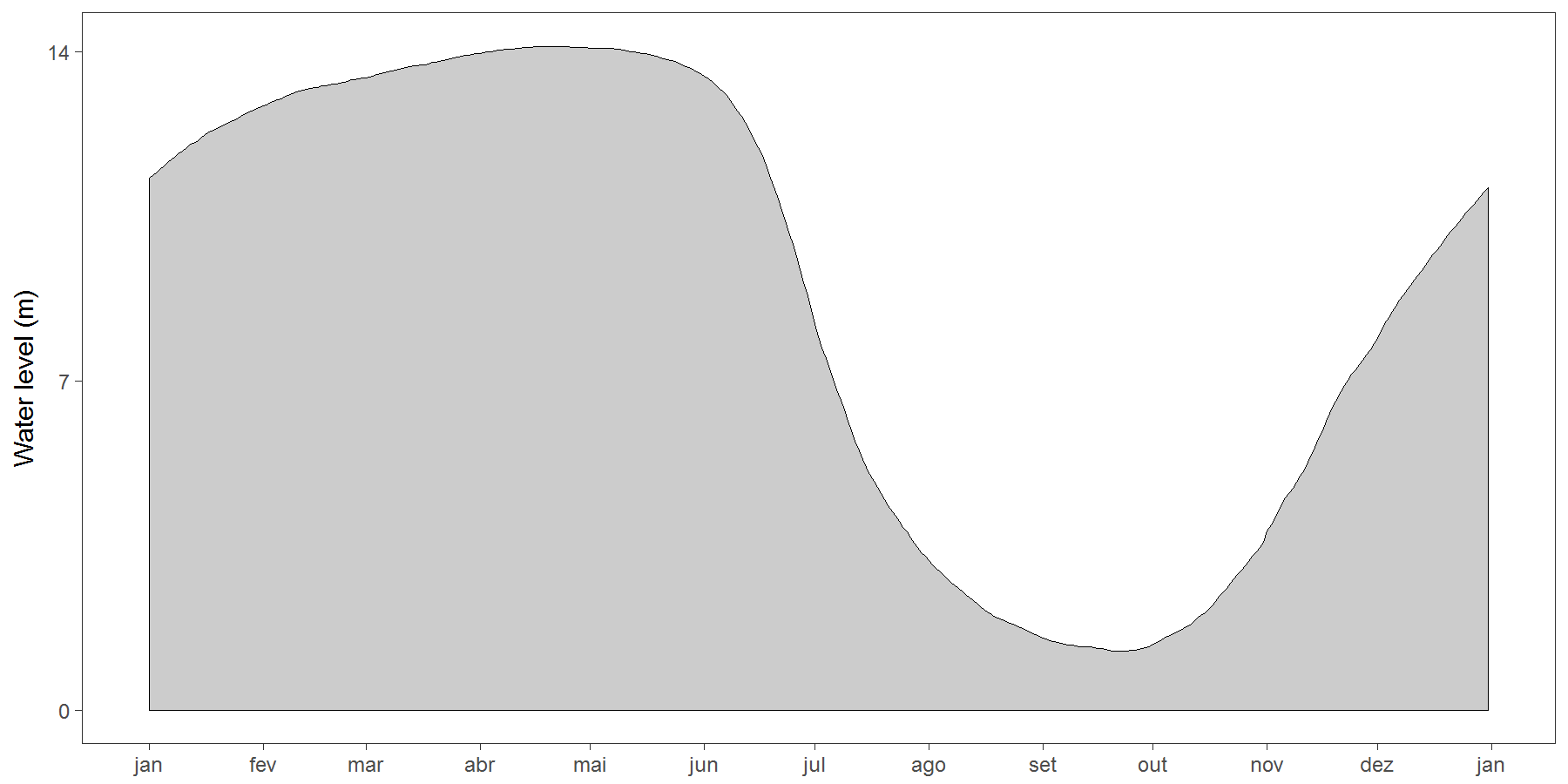

Supplement: Figure S2 [file peerj-06-5058-s006.png]
